# Postoperative atrial fibrillation in patients undergoing coronary artery bypass grafting or cardiac valve surgery: intraoperative use of landiolol

**DOI:** 10.1186/1749-8090-8-19

**Published:** 2013-01-24

**Authors:** Kazuhiro Nakanishi, Shinhiro Takeda, Chol Kim, Shusuke Kohda, Atsuhiro Sakamoto

**Affiliations:** 1 Department of Anesthesiology, Nippon Medical School, 1-1-5 Sendagi, Bunkyo-ku, Tokyo, 113-8603, Japan

**Keywords:** β-blocker, Landiolol, Postoperative atrial fibrillation, Cardiac surgery

## Abstract

**Background:**

Landiolol hydrochloride is a new β-adrenergic blocker with a pharmacological profile that suggests it can be administered safely to patients who have sinus tachycardia or tachyarrhythmia and who require heart rate reduction. This study aimed to investigate whether intraoperative administration of landiolol could reduce the incidence of atrial fibrillation (AF) after cardiac surgery.

**Methods:**

Of the 200 consecutive patients whose records could be retrieved between October 2006 and September 2007, we retrospectively reviewed a total of 105 patients who met the inclusion criteria: no previous permanent/persistent AF, no permanent pacemaker, no renal insufficiency requiring dialysis, and no reactive airway disease, etc. Landiolol infusion was started after surgery had commenced, at an infusion rate of 1 μg/kg/min, titrated upward in 3–5 μg/kg/min increments. The patients were divided into 2 groups: those who received intraoperative β-blocker therapy with landiolol (landiolol group) and those who did not receive any β-blockers during surgery (control group). An unpaired *t* test and Fisher’s exact test were used to compare between-group differences in mean values and categorical data, respectively.

**Results:**

Seventeen of the 105 patients (16.2%) developed postoperative atrial fibrillation: 5/57 (8.8%) in the landiolol group and 12/48 (25%) in the control group. There was a significant difference between the two groups (P=0.03). The incidence of AF after valve surgery and off-pump coronary artery bypass grafting was lower in the landiolol group, although the difference between the groups was not statistically significant.

**Conclusions:**

Our retrospective review demonstrated a marked reduction of postoperative AF in those who received landiolol intraoperatively. A prospective study of intraoperative landiolol for preventing postoperative atrial fibrillation is warranted.

## Background

Atrial fibrillation (AF) is the most common arrhythmic complication after cardiac surgery, with an incidence of approximately 20% to 50% that has not changed despite improvements in anesthesia, surgical techniques, and drug therapy [1]. This is probably due to co-morbidities including older age, left ventricular dysfunction, chronic pulmonary disease, and renal insufficiency, which are also contributors to postoperative AF [2,3]. AF has also been associated with a complicated postoperative course, increased incidence of stroke, increased intensive care unit and total hospital stays, and increased health care costs [2-5].

Prophylaxis with preoperative β-blocker therapy [1], preoperative amiodarone [6], and postoperative atrial pacing [7] has had varying degrees of clinical impact, but recent reviews reflect a growing consensus in favor of prophylactic administration of β-blockers [8,9]. The latest American College of Chest Physicians guidelines for the prevention and management of postoperative AF after cardiac surgery recommended that strong consideration should be given to the prophylactic administration of β-blockers as means of lowering the incidence of new-onset post-cardiac surgery AF (strength of recommendation, A; evidence grade, fair, net benefit, substantial) [8]. However, the role of intraoperative β-adrenergic blockers in preventing postoperative AF is still unknown.

Landiolol hydrochloride is a new β-adrenergic blocker synthesized by Ono Pharmaceutical Co. (Osaka, Japan) that resembles esmolol, but has a greater β1-selectivity and a shorter elimination half-life [10,11]. Landiolol is metabolized very quickly by serum pseudocholinesterase and carboxyesterase in the liver to an inactive metabolite with a half-life of 4 min in healthy human subjects [10]. Renal and hepatic clearance do not contribute to the pharmacokinetics at clinical concentrations, which makes landiolol titratable, and the onset and offset of action are rapid [10]. Landiolol has a higher β1-selectivity than any currently available β-blocker, but has neither intrinsic sympathomimetic activity nor significant membrane-stabilizing activity [10-13]. Landiolol may suppress ventricular and supraventricular arrhythmias [13-15]. The superior pharmacological profile of landiolol may allow for safer use in patients in the acute phase of severe heart disease and in other clinical settings [15]. Therefore, we undertook a retrospective, single-institution study to determine if intraoperative administration of landiolol reduces the incidence of AF after cardiac surgery.

## Methods

We reviewed the records of 200 consecutive patients who underwent elective coronary and/or valve surgery at our institution between October 2006 and September 2007, and who were managed by senior anesthesiologists and cardiologists. (This should signify that the subjects were patients who underwent cardiac surgery performed by surgeons from the same cardiac surgery department, in succession, during a one-year period between October 2006 and September 2007). Patients with previous permanent/persistent AF, a permanent pacemaker, renal insufficiency requiring dialysis and reactive airway diseases were excluded from the analysis, as were those who received amiodarone as initial therapy for AF before initiation of β-blocker therapy. Patients with prior AF were not excluded if they presented for surgery in sinus rhythm without amiodarone therapy. A total of 105 patients met these criteria for review. Infusions of landiolol were started after the beginning of surgery, at the discretion of the attending senior anesthesiologist. Infusion of landiolol was initiated at an infusion rate of 1 μg/kg/min and titrated upward in 3–5 μg/kg/min increments based on heart rate (HR) and blood pressure responses. The patients were divided into 2 groups: those who received intraoperative β-blocker therapy with landiolol (landiolol group) and those who did not receive any β-blockers during surgery (control group). After surgery, each patient was admitted to the intensive care unit (ICU) and was subsequently transferred to the ward. The patients were continuously monitored during the first 72 h after surgery, using bedside monitors that were alarm-triggered. Thereafter all patients were monitored routinely with an alarm-triggered three lead telemetry system until the morning of postoperative day 7. Postoperative β-blocker therapy (landiolol, carvedilol or atenolol) was used at the discretion of the attending senior anesthesiologists and cardiologists in the ICU. Patients with and without diabetes with persistently elevated serum glucose (>180 mg/dL) received IV insulin infusions to maintain serum glucose <180 mg/dL for the duration of ICU care. The serum levels of potassium and other electrolytes were monitored and maintained within the normal range. An unpaired *t* test and Fisher’s exact test were used for comparison of between-group differences in mean values and categorical data, respectively. This study was approved by the ethics committee of Nippon Medical School. The committee has established that retrospective studies involving analysis of medical records can be conducted without informed consent from the subjects.

## Results

Clinical characteristics and perioperative data are shown in Table 1. There were no statistically significant differences between the landiolol and control groups in age, New York Heart Association (NYHA) class, history of hypertension, type of surgery, ejection fraction or preoperative β-blocker use. Overall, 17 of 105 patients (16.2%), 5 of 57 (8.8%) in the landiolol group and 12 of 48 (25%) in the control group, developed postoperative AF (P = 0.03). In the landiolol group, AF developed in 2 patients on postoperative day 2, in 1 patient on postoperative day 3, and in 2 patients on postoperative day 4. The mean duration of landiolol administration was 23.9 ± 32.0 h. In addition, the time of AF onset after admission to the ICU was 3.0 ± 1.2 days in the intraoperative landiolol group, and 2.2 ± 1.0 days in the group that did not receive landiolol. Although the difference was not significant, onset tended to be delayed in the intraoperative landiolol group. Postoperative β-blockers were more frequently used in the landiolol group than in the control group (P < 0.0001). The patients in the landiolol group showed a trend toward shorter times to extubation and length of ICU stay, but the differences between the groups were not statistically significant. There were no differences between the groups in blood glucose or potassium levels, and there were no significant differences in postoperative complications.

**Table 1:** Clinical characteristics and perioperative data for the landiolol and control groups

	**Control**	**Landiolol**	**P value**
	**(n=48)**	**(n=57)**	
Age (yr)	65.7 ± 12.6	67.2 ± 11.5	NS
NYHA class	1.9 ± 0.7	1.9 ± 0.7	NS
BMI	23.0 ± 4.1	23.0 ± 3.4	NS
Hypertension	38 (79.2)	47 (82.4)	NS
Diabetes	14 (29.2)	18 (31.6)	NS
Hyperlipidemia	18 (37.5)	25 (43.9)	NS
Previous CHF	22 (45.8)	23 (40.4)	NS
Previous MI	7 (14.6)	12 (21.1)	NS
Previous AF	8 (16.7)	10 (17.5)	NS
LV Ejection fraction (%)	60.5 ± 14.7	56.4 ± 15.2	NS
Preoperative ACE inhibitor use	38 (79.2)	37 (64.9)	NS
Preoperative β-blocker use	24 (50.0)	29 (50.9)	NS
Postoperative β-blocker use	16 (33.3)	41 (71.9)	<0.0001
Preoperative Cr (mg/dL)	1.1 ± 1.8	1.3 ± 1.6	NS
Preoperative % VC	95 ± 21	94 ± 19	NS
Preoperative FEV 1.0%	82 ± 15	82 ± 14	NS
On-pump CABG	5 (10.4)	9 (15.8)	NS
Off-pump CABG	14 (29.2)	27 (47.4)	NS
Valve surgery			
AVR	16 (33.3)	12 (21.1)	NS
MVR	3 (6.3)	2 (3,5)	NS
MVP	9 (18.8)	6 (10.5)	NS
TVP	1 (2.1)	1 (1.8)	NS
Operating time (min)	344 ± 78	334 ± 86	NS
Blood loss (mL)	1159 ± 1036	1197 ± 1246	NS
Fluid and blood infusion volume (mL)	5471 ± 1657	5405 ± 1948	NS
Urine volume (mL)	1283 ± 933	1270 ± 948	NS
No. of patients receiving intraoperative remifentanil	32 (66.7)	39 (68.4)	NS
Intraoperative remifentanil dose (μg/kg/min)	0.18 ± 0.06	0.21 ± 0.07	NS
Postoperative AF	12 (25.0)	5 (8.8)	0.02
Time of onset of new AF (d)	2.2 ± 1.0	3.0 ± 1.2	NS
Length of ICU stay (d)	3.5 ± 2.0	3.0 ± 2.1	NS
Time to extubation (hr)	20.9 ± 41.6	16.0 ± 29.1	NS
Postoperative complications			
Pulmonary edema	6 (12.5)	7 (12.3)	NS
Stroke	1 ((2.1)	0	NS
Seizures	1 (2.1)	1 (1.8)	NS
Renal failure	0	1 (1.8)	NS

Values are shown as numbers (%) or as the mean ± SD. The unpaired *t*-test was used for comparison of between-group differences in means. Fisher’s exact test was used for comparison of between-group differences in categorical data. *NYHA*: New York Heart Association, *CHF*: congestive heart failure, *MI*: myocardial infarction, *LV*: left ventricular, *ACE*: angiotensin-converting enzyme, *Cr*: creatinine, *AF*: atrial fibrillation, *ICU*: intensive care unit.

Intraoperative systolic blood pressure (SBP), diastolic blood pressure (DBP), and HR are shown in Table 2. In the landiolol group SBP was significantly higher at the start of the operation and SBP and HR were significantly higher at sternotomy, compared to the control group. The incidences of AF after valve surgery and off-pump coronary artery bypass grafting (CABG) were lower in the landiolol group (Table 3), but the differences between the groups were not statistically significant. The percentage incidence of postoperative AF was similar among all operative procedures (Table 3).

**Table 2:** Systolic and diastolic blood pressure and heart rate in the two groups

	**Group**	**Before induction**	**After induction**	**Start of operation**	**Sternotomy**	**End of operation**
SBP (mm Hg)	Control	136 ± 18	88 ± 12	115 ± 19	128 ± 15	115 ± 19
	Landiolol	139 ± 24	93 ± 13	120 ± 19	137 ± 18*	118 ± 19
DBP (mm Hg)	Control	69 ± 16	44 ± 7	56 ± 11	63 ± 12	54 ± 9
	Landiolol	72 ± 17	46 ± 10	58 ± 14	65 ± 15	56 ± 11
HR (beats/min)	Control	68 ± 12	54 ± 10	59 ± 11	65 ± 8	76 ± 9
	Landiolol	70 ± 12	58 ± 13	63 ± 11*	71 ± 12*	75 ± 15

Values are shown as the mean ± SD. *P<0.05 vs. control (unpaired *t*-test). *SBP*: systolic blood pressure, *DBP*: diastolic blood pressure, *HR*: heart rate.

Repeated measures ANOVA showed significant differences between the groups for SBP (p=0.03) and HR (p=0.02). *SBP* was significantly higher in the landiolol group at the start of the operation. *SBP* and *HR* were significantly higher in the landiolol group at sternotomy. *DBP* was similar in both groups.

**Table 3:** Comparison of the incidence of postoperative atrial fibrillation between the landiolol and control groups

	**Total population**	**Control**	**Landiolol**	**P value**
Valve surgery	9/50 (18%)	7/29 (24%)	2/21 (10%)	0.18
On-pump CABG	2/14 (14%)	1/5 (20%)	1/9 (11%)	0.65
Off-pump CABG	6/41 (15%)	4/14 (29%)	2/27 (7%)	0.07
All surgery	17/105 (16%)	12/48 (25%)	5/57 (8.8%)	0.02

Fisher’s exact test was used to analyze between-group differences in categorical data. *CABG*: coronary artery bypass grafting.

The data in Table 4 suggest a lower incidence of postoperative AF in patients who received postoperative β-blocker therapy (12.3%, 7 of 57 patients) compared with those who did not receive this therapy (20.8%, 10 of 48 patients). However, this difference was not statistically significant. Of 57 patients in the landiolol group, β-blocker therapy (landiolol, carvedilol or atenolol) was continued postoperatively in 41 and discontinued after surgery in 16. There was no significant difference in the occurrence of postoperative AF between these sub-groups (9.8% vs. 6.3%, respectively). Patients who received β-blocker therapy only after surgery had an incidence of postoperative AF of 18.8% (3 of 16). A higher rate of AF was found in patients who did not receive β-blockers perioperatively (28.1%, 9 of 32), but the occurrence of postoperative AF did not differ significantly among all the sub-groups.

**Table 4:** Effect of perioperative β-blockers on the incidence of postoperative AF

**Intraoperative β-blocker use**	**Postoperative β-blocker use**	**Incidence of postoperative AF**
(+)	(+)	4/41 (9.8%)
(+)	(−)	1/16 (6.3%)
(−)	(+)	3/16 (18.8%)
(−)	(−)	9/32 (28.1%)

*AF*: atrial fibrillation. There were no significant differences in the incidence of postoperative AF among the groups. Four of the 41 patients who received intraoperative and postoperative β-blockers developed postoperative AF. Two of these patients developed AF after discontinuation of postoperative β-blocker therapy.

## Discussion

AF is a common complication after cardiovascular surgery and often results in prolonged hospital stays and increased morbidity compared with patients who maintain sinus rhythm [2-5]. Approaches to preoperative and postoperative prophylaxis of AF using selective β-blockers [1,8,9], amiodarone [6], and atrial pacing [7] have achieved varying degrees of clinical success. However, the role of intraoperative β-adrenergic blockers in preventing postoperative AF is unknown. In the present study, patients who received intraoperative landiolol had a significantly lower incidence of postoperative AF (8.8%).

In a study of patients who underwent on-pump CABG, an inhibitory effect on postoperative AF was reported from administering landiolol for 48 h from the start of surgery [16]. The results of our study, which includes not only on-pump CABG, but also off-pump CABG and heart valve surgical patients, suggest that AF occurrences can be suppressed even in these cases. Sezai et al. [16] reported that postoperative AF was reduced by treatment with landiolol in patients undergoing CABG on cardiopulmonary bypass (10%, 7 of 70 patients). Landiolol infusion was started at the time of central anastomosis during CABG and discontinued after 48 h. In our study, of 57 patients in the landiolol group, β-blocker therapy was continued postoperatively in 41 patients and discontinued after surgery in 16. The incidence of postoperative AF in patients receiving intraoperative and postoperative β-blocker therapy was 9.8%, which was similar to the values in their study. Similarly, the incidence of postoperative AF in patients who received landiolol only during surgery was 6.3%. Most known risk factors were found to be similar among groups. These results suggest a possible role for prophylactic landiolol during cardiac surgery to reduce the incidence of postoperative AF.

In the postoperative heart, multiple factors may potentially predispose a patient to AF through alterations in refractoriness and/or local reentry. These include operative trauma from surgical dissection and manipulation, inflammation, elevation in atrial pressure from postoperative impaired ventricular function, chemical stimulation during perioperative support with catecholamines and other inotropic agents, reflex sympathetic activation from volume loss, anemia or pain, parasympathetic activation, fever from atelectasis or infection, and hypoglycemia or ischemic damage incurred during surgery [17,18]. Recent studies have shown that markers of oxidative injury and inflammation, such as C-reactive protein, are elevated in patients with AF, indicating that they may have an important role in the pathogenesis of postoperative AF [19,20]. Landiolol has a lipid peroxidation-reducing effect and suppresses the increase in phospholamban serine phosphorylation in the sarcoplasmic reticulum (SR) in hearts subjected to ischemia-reperfusion (I/R) [21]. Landiolol, as well as propranolol and esmolol, also has cardioprotective effects in isolated guinea pig hearts subjected to I/R injury [22]. Therefore, landiolol may have anti-ischemic properties as an antioxidant and via preservation of SR function during the ischemic period. In addition, preischemic administration of landiolol reduced cardiac cellular damage and improved the recovery of cardiac function in the rat heart through protein kinase C (PKC) epsilon-mediated pathway, similar to that afforded by ischemic preconditioning [23]. Reduction of postoperative AF by landiolol may be due to reduced inflammation or oxidative stress, or pharmacological preconditioning effect, in addition to effective β-blockade.

Cardiac surgery requiring cardiopulmonary bypass results in significant impairment of β-adrenergic receptor (βAR) function [24]. This can cause depressed postoperative myocardial function that results in atrial enlargement, which is a risk for postoperative AF. The possible mechanisms underlying impaired βAR function include myocardial ischemia-reperfusion injury [25], a hibernating and stunned myocardium [26], and acute myocardial βAR desensitization [27,28]. A marked increase in catecholamine levels found during cardiac surgery resulting in agonist-induced desensitization may be a major mechanism behind acute βAR desensitization [27]. Increased myocardial catecholamine concentrations may, at least in part, be the stimulus for the myocardial hyporesponsiveness seen during cardiac surgery. In support of this, blockade of βARs with low-dose βAR antagonist therapy improves myocardial function in congestive heart failure [29], a clinical setting in which attenuation of chronic βAR desensitization has been postulated as a possible mechanism. In the landiolol group SBP was significantly higher at the start of the operation and SBP and HR were significantly higher at sternotomy, compared to the control group. These results suggested that serum catecholamine levels in the landiolol group may increase during surgery. Nevertheless, we found a marked reduction of postoperative AF in the landiolol group. Landiolol may have the suppressive effect of catecholamine-induced βAR desensitization. Booth et al. reported that intravenous esmolol administration during coronary artery revascularization after acute myocardial ischemia results in improved left ventricular function immediately after cardiopulmonary bypass (CPB) via enhanced βAR signaling [30], whereas chronic oral ß-adrenergic blocker therapy in patients undergoing CABG surgery does not protect patients from acute βAR desensitization [27]. In addition, Cork et al. demonstrated improved early intermediate outcomes (such as cardiac output) immediately post-CPB in patients receiving an intraoperative β-adrenergic blocker [31]. In a similar manner, improvement of βAR function by intraoperative administration of landiolol may contribute to preserved left ventricular function and fewer inotropic requirements postoperatively, resulting in a reduced incidence of postoperative AF.

The prevalence of AF increases with worsening heart failure from 5% in patients of NYHA class I under drug therapy to as high as 50% in those of NYHA class IV. Structural, electrophysiologic, and neurohumoral mechanisms may play important roles in this effect, but an association of heart failure and AF has not been established in the postoperative setting. Factors predisposing patients to postoperative AF may be unrelated to left ventricular function, and in our study patients who received landiolol intraoperatively experienced less AF than those who did not, despite having similar left ventricular ejection fractions (56.4% vs. 60.5%). However, preoperative left ventricular dysfunction is one of the strongest predictors of mortality after CABG surgery. The use of ultra-short-acting β-blockers has been suggested as a safe and efficacious method for treating patients with accelerated adrenergic drive during and after the postoperative phase of cardiovascular surgery. Landiolol hydrochloride is approximately nine times more potent in β-blocking activity in vivo and eight times more cardioselective in vitro than esmolol [10]. Moreover, the suppressive effects on cardiovascular perforance are significantoly less potent or landiolol than those of esmolol at equipotent β-blocking doses [13]. These properties facilitate a more rapid recovery after drug administration if an adverse effect develops, which should lead to safer perioperative management of patients undergoing cardiac surgery and in other clinical settings.

Our study had several linitations. First, our patients had undergone various cardiac procedures with or without the use of a heart-lung machine. Second, they were not randomly assigned to either group and there was a between-group difference in the number and type of the cardiac surgery that the patients had undergone. Third, the optimum dose for intraoperative landiolol could not be verified, including in our investigations. Therefore, to confirm the preventive effect of intraoperative landiolol on AF after cardiac surgery, a large-scale clinical study is needed.

The main points of our findings are: (1) when landiolol was not administered intraoperatively, the incidence of postoperative AF was lower in the group that received postoperative β-blockers than in the group that did not; (2) even when postoperative β-blockers were used, the incidence of postoperative AF was lower in the group that received intraoperative landiolol; and (3) when landiolol was administered intraoperatively, the incidence of postoperative AF was low regardless of whether or not postoperative β-blockers were used.

## Conclusions

Although this was a retrospective, nonrandomized study, our retrospective review of 105 patients who underwent cardiac surgery demonstrated a marked reduction in postoperative AF (8.8%) in those who received landiolol during surgery. In the future we expect to conduct a randomized prospective study to investigate the inhibitory effects of intraoperative landiolol on postoperative AF, in patients who have undergone off-pump CABG and heart valve surgery.

## Abbreviations

AF: Atrial fibrillation; βAR: β-adrenergic receptor; CABG: Coronary artery bypass grafting; CPB: Cardiopulmonary bypass; DBP: Diastolic blood pressure; HR: Heart rate; ICU: Intensive care unit; NYHA: New York Heart Association; SBP: Systolic blood pressure.

## Competing interests

The authors declare that they have no competing interests.

## Authors’ contributions

All authors have made substantial contribution to study conception and design, acquisition and analysis of data, and manuscript preparation, and have approved the final manuscript.
